# Four-Dimensional Cine Cinematic Rendering of Structural Heart and Mechanical Circulatory Support Devices: An Illustrative Technical Experience

**DOI:** 10.3390/jimaging12080390

**Published:** 2026-08-19

**Authors:** Amy Avakian, Muhammad Umair

**Affiliations:** 1 Southern Hills Hospital and Medical Center, Las Vegas, NV 89148, USA; 2 Department of Radiology, Columbia University Irving Medical Center, New York, NY 10032, USA; mu2331@cumc.columbia.edu; 3 Russell H. Morgan Department of Radiology and Radiological Sciences, Johns Hopkins University, Baltimore, MD 21287, USA

**Keywords:** cinematic rendering, 4D cine cardiac CT, cardiac implants, mechanical circulatory support, transcatheter heart valves, photorealistic visualization

## Abstract

Patients with implanted cardiac devices are a rapidly growing imaging population, and electrocardiogram-gated cardiac computed tomography (CT) is increasingly used to characterize device geometry, multi-device relationships, and dynamic behavior across the cardiac cycle. Cinematic rendering (CR) is a photorealistic three-dimensional (3D) visualization technique for cardiac CT whose established contribution in this population is communicative: it conveys 3D device geometry and material distinctions within a single rendered volume. We describe a demonstrative case series extending CR across the cardiac cycle—time-resolved “4D cine” CR—to depict dynamic device behavior and time-resolved multi-device interaction in a single volume; this is an illustrative technical experience rather than a systematic evaluation of diagnostic performance. Illustrative examples include an EVOQUE transcatheter tricuspid valve rendered together with concurrent surgical mitral and transcatheter aortic valves, a left atrial appendage occlusion device, a normally positioned Impella catheter, and a HeartMate 3 left ventricular assist device (LVAD). Across cases, 4D cine CR feasibility scaled inversely with metallic burden—the aggregate volume and radiodensity of metallic device components within the scan field—with renderings informative for low-metal nitinol and catheter devices but substantially degraded by streak artifact in high-metal LVAD housings. This relationship was observed qualitatively in a small selected series and is offered as an initial observation rather than an established characteristic of the technique. We discuss current limitations and emerging directions such as photon-counting detector CT, metal artifact reduction, and artificial-intelligence-assisted post-processing that may extend 4D cine CR in this population.

## 1. Introduction

Patients with implanted cardiac devices are a rapidly growing imaging population. Continuous-flow left ventricular assist devices (LVADs) are increasingly used as destination therapy [1], transcatheter aortic valve replacement (TAVR) has expanded into low-risk cohorts [2], and the Impella platform (Abiomed, Danvers, MA, USA) is widely deployed for cardiogenic shock and high-risk percutaneous coronary intervention [3]. The structural heart landscape now also includes transcatheter mitral and tricuspid valve replacement (TMVR, TTVR) and left atrial appendage occlusion (LAAO) devices, all increasingly encountered on cross-sectional imaging. Radiologists are frequently asked to evaluate patients carrying several devices simultaneously, with failure modes including cannula malposition, outflow-graft compression, prosthetic leaflet thrombosis, and dynamic device interaction across the cardiac cycle [4,5,6,7]. Electrocardiogram (ECG)-gated cardiac computed tomography (CT) is central to this assessment.

Cinematic rendering (CR) uses Monte Carlo path tracing with global illumination to produce photorealistic surface gradients and depth cues that more closely approximate natural visual perception [8,9]. Compared with traditional volume rendering, CR has been rated favorably for depth perception and delineation of structures adjacent to the heart [10], and its principles and applications in cardiac CT—including the black-blood CR variant for endoluminal visualization—have been detailed [11,12]. To date, CR of cardiac devices has largely been applied to single, static 3D volumes, where its contribution is communicative rather than diagnostic.

Recently, CR has been extended across the cardiac cycle by rendering each phase of a retrospectively gated acquisition, an approach termed time-resolved or “4D cine” CR. This opens a potential window onto dynamic device behavior—leaflet excursion, cannula–wall contact, and time-resolved multi-device relationships—within a single rendered volume. Here we describe a demonstrative case series applying 4D cine CR across a range of structural heart and mechanical circulatory support (MCS) devices, report an initial observed relationship between rendering feasibility and device metallic burden—defined here as the aggregate volume and radiodensity of metallic device components within the scan field—and outline current limitations and future directions. This report concerns visualization technique and is explicitly not a systematic evaluation of diagnostic performance: it does not assess diagnostic accuracy or reader performance, includes no comparison arm, and CR is not proposed as a substitute for conventional two-dimensional (2D) reformats, echocardiography, or device-specific surveillance. The cases shown were selected to illustrate the range of rendering behavior across device types and do not constitute a consecutive or representative sample.

## 2. Materials and Methods

### 2.1. Study Design and Case Selection

This is a retrospective, single-center demonstrative case series. Five patients with implanted structural heart or mechanical circulatory support devices who had undergone clinically indicated, retrospectively ECG-gated cardiac CT angiography (CTA) were identified, and their fully de-identified datasets were used for time-resolved cinematic rendering. Cases were selected purposively to span a range of device types and metallic composition—a transcatheter tricuspid valve with concurrent surgical mitral and transcatheter aortic prostheses, a left atrial appendage occlusion device, a percutaneous microaxial support catheter, and two durable left ventricular assist devices—rather than consecutively. No imaging was performed for the purposes of this report, and no patient received additional radiation exposure as a result of it.

### 2.2. CT Acquisition

All examinations were acquired on a dual-source 192-slice scanner (SOMATOM Force; Siemens Healthineers, Forchheim, Germany) with a 2 × 192 × 0.6 mm detector configuration using a z-flying focal spot. Acquisitions were performed at 120 kV on both tube A and tube B, with automated tube current modulation (CARE Dose4D) at a reference of 320 mAs, a gantry rotation time of 0.25 s, and a pitch of 0.15 adapted for ECG-gated acquisition. Scanning was craniocaudal, extending from the thoracic inlet to below the diaphragm so as to include the outflow graft anastomosis to the ascending aorta and the inflow cannula at the left ventricular apex in device-bearing patients. Retrospective ECG gating was used with padding across the full R–R interval (0–100%), and ECG editing was applied as needed for ectopy.

### 2.3. Contrast Protocol

Iohexol (Omnipaque 350; 350 mg I/mL) was administered as an 80 mL bolus at 5 mL/s through an 18-gauge antecubital intravenous cannula, with the right arm preferred, followed by a 40 mL saline chaser at the same rate. Acquisition was triggered by bolus tracking with a region of interest positioned in the descending aorta, a threshold of 150 HU, and an 8 s delay after trigger.

### 2.4. Image Reconstruction

Ten phases were reconstructed per cardiac cycle at 10% increments across the R–R interval (0%, 10%, 20% … 90%). Images were reconstructed with a vascular medium-smooth kernel (Bv40) for soft-tissue evaluation and a vascular medium-sharp kernel (Bv60) for device and hardware assessment, using advanced modeled iterative reconstruction (ADMIRE) at strength 3. The primary series was reconstructed at 0.75 mm slice thickness with a 0.5 mm increment; an axial review series was reconstructed at 3.0 mm thickness and increment. The field of view was 300 mm, adjusted to patient habitus.

### 2.5. Cinematic Rendering Post-Processing

Cinematic rendering was performed on a dedicated post-processing workstation using Cinematic VRT (syngo.via VB30; Siemens Healthineers, Forchheim, Germany). Each of the ten reconstructed cardiac phases was rendered independently, and the resulting volumes were assembled into a time-resolved cine loop, yielding the 4D cine CR series. Approximate operator time to generate a single 4D cine CR model, including phase loading, preset selection, cut-plane definition, and camera positioning, was 15 min per case. Rendering presets are vendor-specific and are not transferable between platforms, which limits exact reproduction of these renderings on other systems.

### 2.6. Radiation Dose

For a representative retrospectively gated examination performed with this protocol, the volume CT dose index was 38.2 mGy and the dose–length product was 1074 mGy·cm, corresponding to an estimated effective dose of approximately 15.0 mSv using a chest conversion factor of k = 0.014 mSv/(mGy·cm). Retrospective gating with full R–R padding carries a higher dose than prospectively triggered protocols, and is justified in these patients only where assessment across the cardiac cycle is clinically required—for example, evaluation of prosthetic leaflet motion, dynamic cannula–wall contact, or suspected device-related obstruction. Where such dynamic information is not needed, a prospectively triggered acquisition remains appropriate, and 4D cine CR is correspondingly not available.

## 3. Three-Dimensional Cinematic Rendering: Established Communicative Role

**Figure 1:**
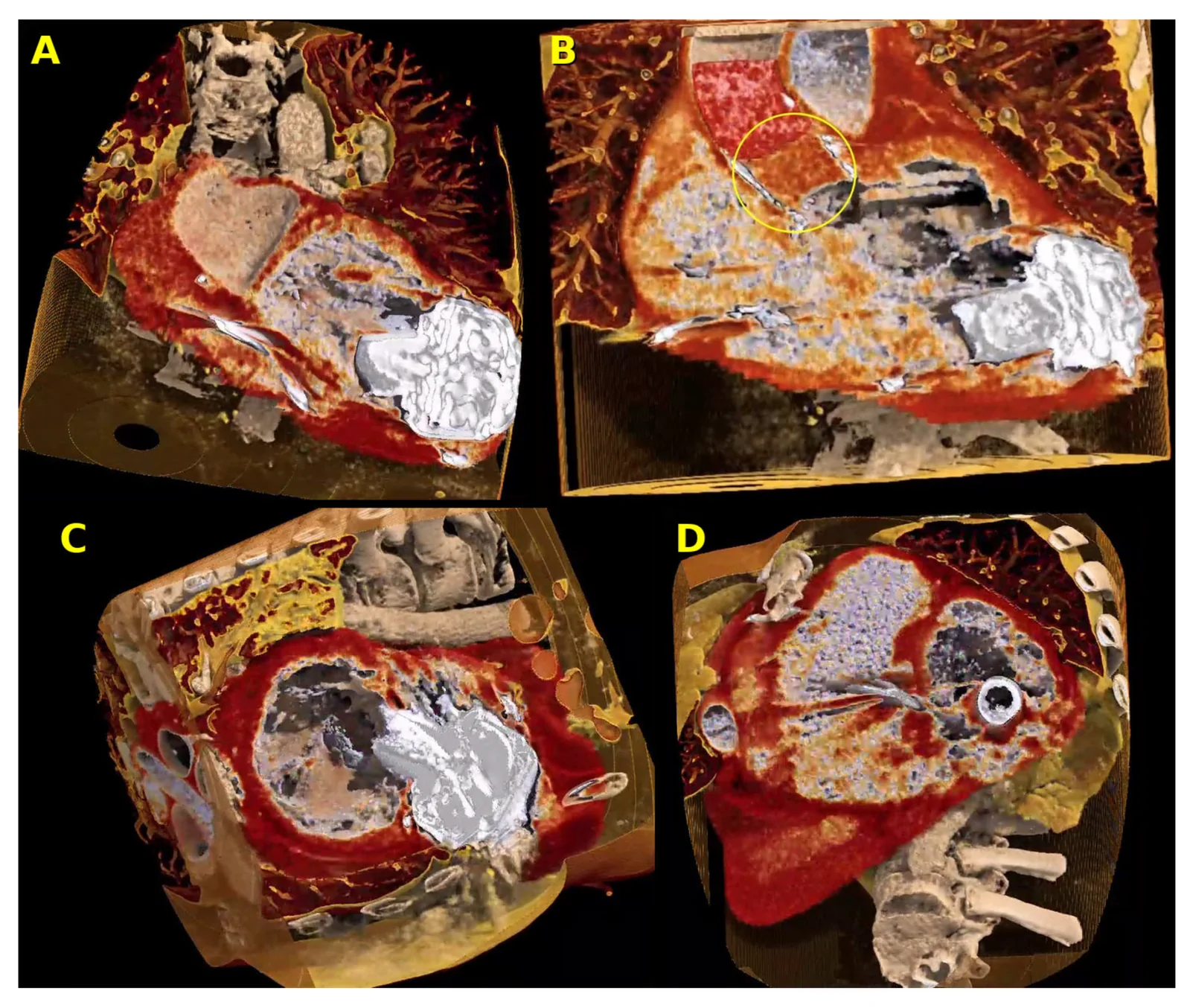
Cinematic rendering (CR) in two patients with mechanical circulatory support. (**A**,**B**) Patient with a concurrent HeartMate 3 LVAD and prior TAVR. (**A**) Cut-surface, off-center two-chamber 3D CR showing eccentric positioning of the LVAD inflow cannula toward the apical inferior wall rather than the ideal mitral-valve-directed orientation. (**B**) 3D CR at the level of the TAVR prosthesis (three-chamber view) with leaflet thrombosis (yellow circle) extending above the leaflet tips; material-specific color mapping conveys the spatial extent of thrombus. (**C**,**D**) Separate patient with a durable LVAD (no TAVR) in whom 4D cine CR was attempted. (**C**) Short-axis 4D cine CR of the LVAD pump housing and surrounding myocardium across the cardiac cycle (Supplementary Video S5). (**D**) 4D cine CR showing streak artifact from metallic LVAD components, a current technical limitation (Supplementary Video S6). LVAD, left ventricular assist device; TAVR, transcatheter aortic valve replacement.

The established contribution of single-volume 3D CR in this population is efficient communication of device geometry, orientation, and the relationship of devices to adjacent anatomy already characterized on 2D reformats. Prior work reported the value of CR in distinguishing external biodebris compression from intraluminal thrombus as the cause of LVAD outflow-graft narrowing [4]. Figure 1 extends that experience to a patient with a concurrent HeartMate 3 LVAD (Abbott, Chicago, IL, USA) and prior TAVR. CR depicts the eccentric LVAD inflow cannula directed toward the apical inferior wall rather than the ideal mitral-valve-directed orientation (Figure 1A). Inflow-cannula geometry has been associated with pump function and thrombosis risk [5], and tip-to-wall contact during systole can produce dynamic obstruction; equivalent assessment on 2D CTA requires multiple dedicated reformats. On the same examination, CR depicted thrombosis of the TAVR leaflets extending above the leaflet tips (Figure 1B); material-specific color mapping conveys the spatial extent of thrombus across the prosthesis in a single view [6,7]. In high-metal devices such as LVADs, CR does not reduce streak and beam-hardening artifacts and may accentuate them [13].

## 4. Four-Dimensional Cine Cinematic Rendering: Illustrative Experience

Time-resolved 4D cine CR renders each phase of a retrospectively gated CTA acquisition, allowing structural depiction and cross-cycle behavior to be viewed within a single dataset: dynamic cannula–wall interaction during systole, prosthetic leaflet excursion, and time-resolved relationships between adjacent devices. The cases below illustrate the range of rendering behavior encountered across device types.

### 4.1. Transcatheter Tricuspid Valve and Multi-Device Rendering

**Figure 2:**
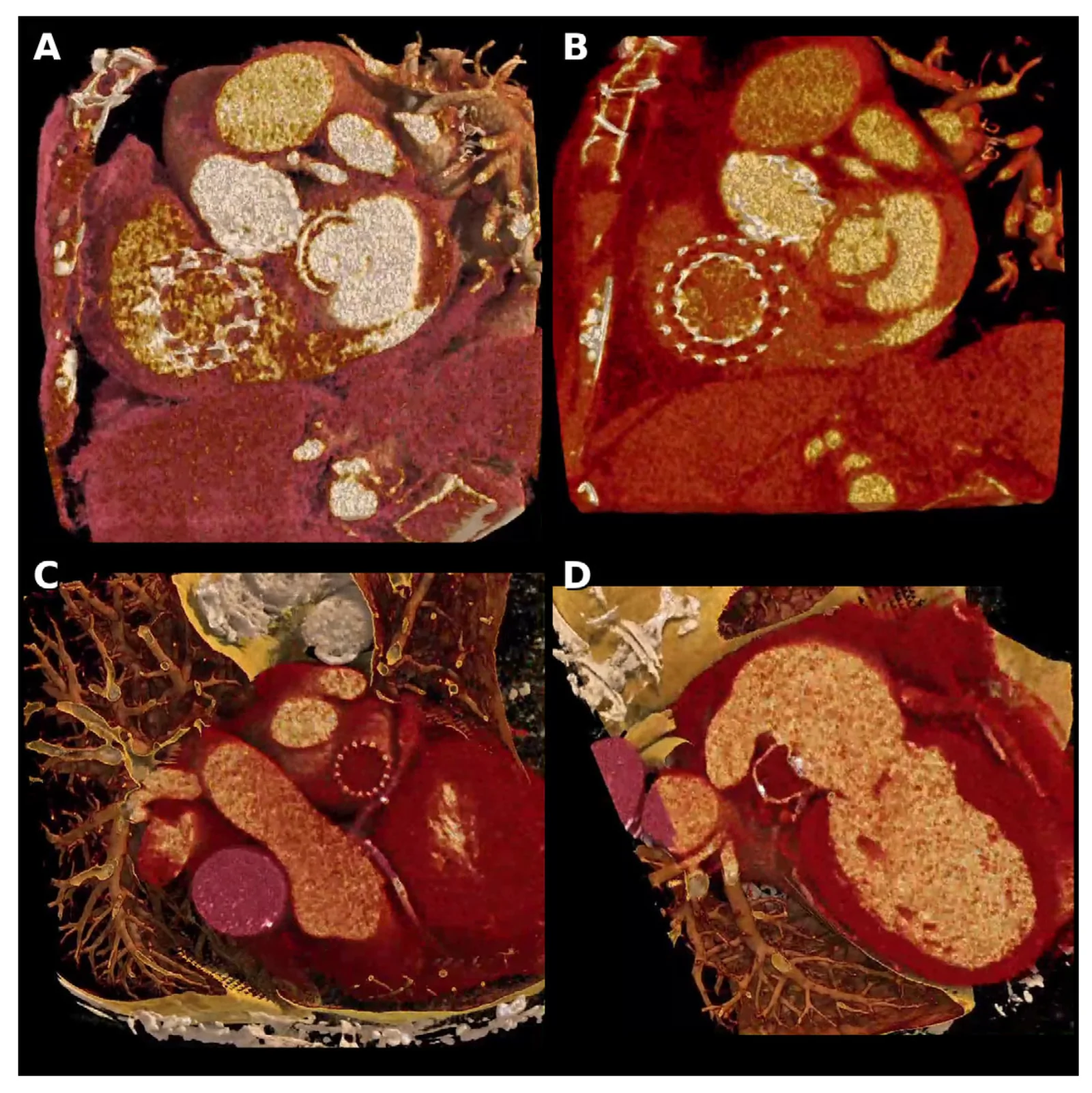
Four-dimensional (4D) cine CR extending the technique to transcatheter tricuspid valve replacement and left atrial appendage occlusion (LAAO). (**A**,**B**) Patient with an EVOQUE transcatheter tricuspid valve, concurrent surgical mitral valve, and prior TAVR. (**A**) 4D cine CR at an en-face level through the EVOQUE prosthesis showing markedly thickened leaflets with restricted mobility across the cardiac cycle (Supplementary Video S1). (**B**) 4D cine CR depicting all three prostheses—EVOQUE tricuspid, surgical mitral, and TAVR—within a single rendered volume (Supplementary Video S2). (**C**,**D**) Separate patient with a deployed LAAO device. (**C**) Wide-context 4D cine CR showing the LAAO device in relation to the left circumflex artery and mediastinal structures across the cardiac cycle. (**D**) Focused cut-surface 4D cine CR showing the longitudinal central cross-section of the LAAO device and sealing of the left atrial appendage (Supplementary Video S3).

Dynamic leaflet behavior is central to transcatheter valve surveillance. In a patient with an EVOQUE transcatheter tricuspid valve replacement (Edwards Lifesciences, Irvine, CA, USA), an en-face 4D cine CR view depicted markedly thickened prosthetic leaflets with restricted mobility across the cardiac cycle (Figure 2A; Supplementary Video S1). In the same patient, a single 4D cine reconstruction simultaneously rendered the EVOQUE prosthesis, a surgical mitral valve, and a TAVR prosthesis within one volume across the cardiac cycle (Figure 2B; Supplementary Video S2), illustrating the capacity of CR to depict complex multi-device geometry and time-resolved interaction in patients with combined surgical and transcatheter interventions.

### 4.2. Left Atrial Appendage Occlusion

LAAO devices have a predominantly nitinol frame and lower aggregate metallic burden than mechanical pumps. In a patient with a deployed LAAO device, 4D cine CR provided dynamic visualization of device seating within the left atrial appendage ostium, the relationship of the device to surrounding mediastinal and atrial structures, and device–tissue apposition across the cardiac cycle (Figure 2C,D; Supplementary Video S3). The photorealistic depiction conveys device orientation and contact in a single volume, complementary to 2D reformats and transesophageal echocardiography.

### 4.3. Percutaneous Mechanical Circulatory Support (Impella)

**Figure 3:**
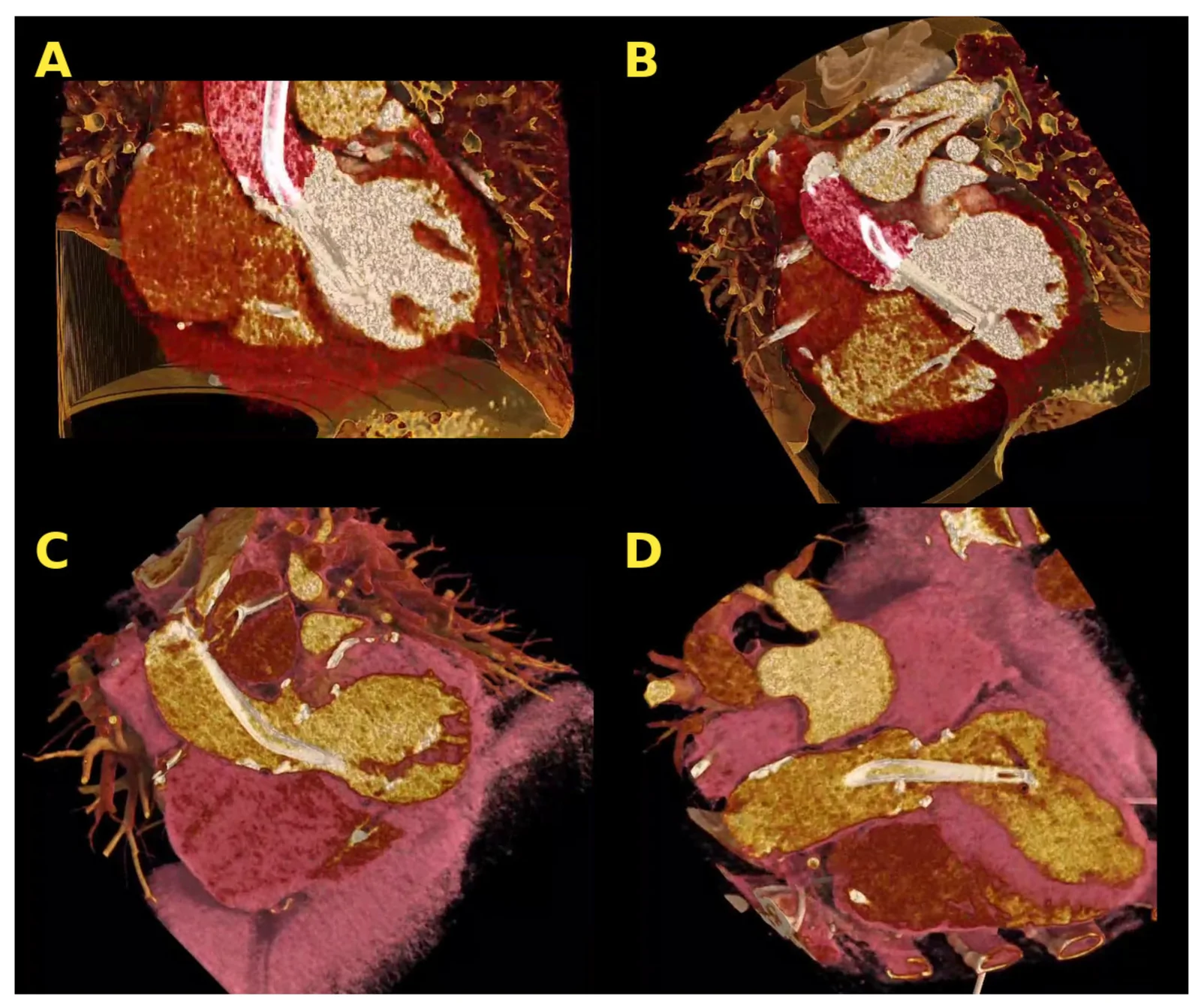
Four-dimensional (4D) cine CR in a patient with a normally positioned Impella device, showing device geometry across the cardiac cycle. (**A**,**B**) Oblique coronal and oblique cut-surface 4D cine renderings emphasizing the catheter trajectory across the aortic valve and the device position within the LV cavity (Supplementary Video S4). (**C**,**D**) 4D cine renderings using an alternative preset emphasizing the aortic root and adjacent structures, with the catheter crossing the aortic valve. LV, left ventricle.

In a separate patient, 4D cine CR was obtained of a normally positioned Impella catheter, with catheter geometry and transaortic trajectory rendered across the cardiac cycle using multiple presets and viewing angles (Figure 3A,B; Supplementary Video S4), and an alternative preset emphasizing the aortic root and adjacent structures (Figure 3C,D). Impella position is identifiable on conventional CTA and routinely assessed clinically [14]; the CR contribution is efficient communication of dynamic device–valve and device–chamber geometry within a single rendered volume. Streak artifact was qualitatively reduced relative to the LVAD examples, consistent with the smaller metallic footprint of the catheter pump [13].

### 4.4. Durable LVAD and the Limits of Feasibility

In a separate patient with a durable LVAD, 4D cine CR provided a short-axis view of the pump housing and surrounding myocardium across the cardiac cycle (Figure 1C; Supplementary Video S5). However, streak artifact from the metallic components substantially degraded image quality in the time-resolved reconstructions (Figure 1D; Supplementary Video S6) and appeared amplified relative to single-phase CR, although a rigorous mechanistic study is not yet available.

### 4.5. Feasibility and Metallic Burden

Across these cases, 4D cine CR feasibility scaled inversely with device metallic burden. Low-metal nitinol frames (LAAO) and catheter-based pumps (Impella) yielded interpretable time-resolved renderings, whereas high-metal durable LVAD housings produced streak artifact that degraded the 4D reconstruction. This relationship was assessed qualitatively in a small, selected series and is presented as an initial observation rather than a general characteristic of the technique. It offers a provisional expectation for when 4D cine CR is likely to add communicative value, and requires systematic quantitative study before it can be generalized.

## 5. Current Limitations

This is a small, single-center demonstrative case series without quantitative or outcome measures, and CR does not provide detection beyond conventional reformats. The cases were selected for their illustrative value, and this selection is likely to favor examples in which the technique performed well; the series should not be read as an unbiased estimate of how often 4D cine CR is informative. The metallic-burden relationship is qualitative. Renderings were assessed visually by the authors; no independent blinded observer scoring was performed, no standardized image-quality scale was applied, and inter-observer agreement was therefore not measured. No quantitative artifact metrics—such as signal-to-noise ratio, contrast-to-noise ratio, or artifact index derived from regions of interest adjacent to the devices—were computed on the source reconstructions, and artifact severity was not compared between systolic and diastolic phases. The relationship between metallic burden and rendering quality is therefore descriptive and not mechanistically characterized. No comparison was made against conventional cine CT, multiplanar reformations, or standard volume rendering, so these data cannot establish whether 4D cine CR conveys information beyond that already available on conventional reconstructions. Cinematic rendering produces a photorealistic image without preserved Hounsfield values, so image-quality metrics cannot be derived from the renderings themselves and must instead be computed on the source reconstructions that feed the rendering pipeline. Time-resolved rendering is computationally intensive, and presets are vendor-specific, limiting exact reproducibility across platforms. The retrospectively gated acquisitions required for 4D cine CR also carry a higher radiation dose than prospectively triggered protocols.

## 6. Future Directions

Several developments may extend the role of 3D and 4D CR in this population. Photon-counting detector CT, with improved spectral information and noise behavior at metal interfaces, is a natural substrate for CR pipelines previously constrained by artifacts [15]. Metal artifact reduction algorithms compatible with CR would expand the reach of 4D cine CR into higher-metal devices. Artificial-intelligence-assisted post-processing—for example, automated device segmentation and parameterization of cannula angle and leaflet excursion—has been proposed as a route toward a quantitative adjunct, although no such application has yet been demonstrated for 4D cine CR and this direction remains speculative. Augmented- and extended-reality applications using CR-derived volumes have been described for surgical planning [16], and the trajectory of structural heart procedures may favor immersive multi-device rehearsal environments. Outcome-linked validation and vendor-agnostic preset standardization are needed before incorporation into surveillance protocols, and continued gains in computational performance will make 4D CR more practical for routine use.

## 7. Conclusions

Cinematic rendering is not a replacement for 2D reformats, echocardiography, or device-specific surveillance, and it does not currently provide detection beyond conventional reformats. Its role in cardiac device imaging is as a communication and characterization adjunct that conveys three-dimensional device geometry, multi-device relationships, and material distinctions within a single rendered volume. In this illustrative experience, extending CR across the cardiac cycle as 4D cine CR added a dynamic, time-resolved window whose feasibility was bounded by device metallic burden. These observations represent an illustrative technical experience rather than validation of a new imaging technique; studies evaluating reader performance, diagnostic accuracy, and clinical impact will be required to establish the value of 4D cine CR in cardiovascular imaging. As photon-counting CT, metal artifact reduction, and AI-assisted post-processing mature, both 3D and 4D CR are positioned for expanded roles in this growing population.

## Data Availability

The original contributions presented in this study are included in the article and Supplementary Material. Further inquiries can be directed to the corresponding author.

## Supplementary Materials

The following supporting information can be downloaded at https://www.mdpi.com/article/10.3390/jimaging12080390/s1. Video S1: 4D en-face cine CR of the EVOQUE tricuspid prosthesis showing thickened leaflets with restricted mobility across the cardiac cycle; Video S2: 4D cine CR of three concurrent prostheses (EVOQUE tricuspid, surgical mitral, and TAVR) rendered in a single volume across the cardiac cycle; Video S3: 4D cine CR of the LAAO device in a focused cut-surface view, showing device–tissue sealing of the left atrial appendage across the cardiac cycle; Video S4: 4D cine CR of the Impella catheter showing the trans-aortic-valve trajectory across the cardiac cycle; Video S5: 4D cine CR of the durable LVAD pump housing and surrounding myocardium in short-axis across the cardiac cycle; Video S6: 4D cine CR of the durable LVAD showing streak artifact from metallic components (the feasibility limit).
